# Randomised controlled trial comparing uptake of NHS Health Check in response to standard letters, risk-personalised letters and telephone invitations

**DOI:** 10.1186/s12889-019-6540-8

**Published:** 2019-02-21

**Authors:** Christopher J. Gidlow, Naomi J. Ellis, Victoria Riley, Tim Chadborn, Amanda Bunten, Zafar Iqbal, Aliko Ahmed, Alistair Fisher, David Sugden, David Clark-Carter

**Affiliations:** 1 Centre for Health and Development (CHAD), Staffordshire University, Brindley Building, Leek Road, Stoke-on-Trent, ST4 2DF England; 2 Public Health England, Skipton House, 80 London Road, London, SE1 6LH England; 3 Midlands Partnership NHS Foundation Trust, St George’s Hospital, Corporation Street, Stafford, ST16 3AG England; 4 Public Health England East of England, Victoria House, Capital Park, Fulbourn, Cambridge, CB21 5XA England; 5 Stoke-on-Trent City Council, Glebe Street, Stoke-on-Trent, ST4 1HH England; 6 Staffordshire County Council, 1 Staffordshire Place, Stafford, ST16 2LP England; 7 Centre for Psychological Research, Staffordshire University, The Science Centre, Leek Road, Stoke-on-Trent, ST4 2DF England

**Keywords:** Vascular disease, Health check, Risk, Prevention, Policy implementation

## Abstract

**Background:**

NHS Health Check is a primary prevention programme offering cardiovascular disease (CVD) risk assessment to adults in England aged 40–74. Uptake remains a challenge and invitation method is a strong predictor of uptake. There is evidence of low uptake when using invitation letters. Telephone invitations might increase uptake, but are not widely used. We explored the potential to improve uptake through personalising letters to patient’s CVD risk, and to compare this with generic letters and telephone invitations.

**Methods:**

HEalth Check TRial (HECTR) was a three-arm randomised controlled trial in nine general practices in Staffordshire (UK). Eligible patients were randomised to be invited to a NHS Health Check using one of three methods: standard letter (control); telephone invitation; letter personalised to the patient’s CVD risk. The primary outcome was attendance/non-attendance. Data were collected on a range of patient- and practice-level factors (e.g., patient socio-demographics, CVD risk, practice size, Health Checks outside usual working hours). Multi-level logistic regression estimated the marginal effects to explore whether invitation method predicted attendance. Invitation costs were collated from practices to estimate cost benefit.

**Results:**

In total, 4614 patients were included in analysis (mean age 50.2 ± 8.0 yr.; 52.4% female). Compared with patients invited by standard letter (30.9%), uptake was significantly higher in those invited by telephone (47.6%, *P* < .001), but not personalised letter (31.3%, *p* = .812). In multi-level analysis, compared with the standard letter arm, likelihood of attendance was 18 percentage points higher in the telephone arm and 4 percentage points higher in the personalised letter arm. The effect of telephone calls appeared strongest in patients who were younger and had lower CVD risk. We estimated per 1000 patients invited, risk-personalised letters could result in 40 additional attended Health Checks (at no extra cost) and telephone invitations could result in 180 additional Health Checks at an additional cost of £240.

**Conclusions:**

Telephone invitations should be advocated to address the substantial deficit between current and required levels of NHS uptake, and could be targeted at younger and lower CVD risk adults. Risk-personalised letters should be explored further in a larger sample of high risk individuals.

**Trial registration:**

Registration number: ISRCTN15840751 date of registration: 24/10/2017.

**Electronic supplementary material:**

The online version of this article (10.1186/s12889-019-6540-8) contains supplementary material, which is available to authorized users.

## Background

Cardiovascular disease (CVD) is the leading cause of mortality in the UK, accounting for 27% of all deaths [1]. NHS Health Check is a national CVD risk assessment programme for adults in England aged 40–74 who do not have existing CVD-related conditions (e.g., diabetes, kidney disease) [2]. Typically, general practice records are used to identify eligible patients who are then invited for a Health Check, usually by letter, but sometimes using telephone invitations (or a combination) and more recently, text messages and email. During the Health Check, practitioners should use the patient’s level of CVD risk, to inform a discussion around risk management and reduction [3].

This study focused on uptake, which is fundamental to the success of such population-based risk identification and management programmes [4, 5], and remains a challenge in NHS Health Check. Nationally, uptake is less than 50%; i.e., less than half of those offered a Health Check receive one [6]. This is below the level on which original modelling for cost-effectiveness was based (75%) [7]. Research exploring predictors of attendance has identified sociodemographic patterning. For example, there is evidence of lower uptake in men, younger eligible adults and those with better health profiles, with mixed evidence for deprivation [5, 8–13] and ethnicity [5, 14, 15]. Qualitative work has offered some insight into why people do not take up the NHS Health Check offer. A recent synthesis of data from qualitative studies identified four common reasons: lack of awareness or knowledge; time constraints or competing priorities; misunderstanding the purpose of the Health Check; an aversion to preventive medicine (e.g., reluctance to identify potential problems) [13]. One of the reviewed studies involved interviews with 41 non-attenders to Health Checks and revealed that approximately one-third of participants did not recall receiving their invitation letter, with others reporting that it lacked relevant information or was not prioritised [16].

Method of invitation has been identified as a strong predictor of uptake. Analysis of data from a small number of general practices suggested that use of verbal or telephone invitations yielded uptake that was approximately three-times higher than postal invitations [11]. Others have similarly found that use of telephone calls elicited better uptake in Health Checks. Cook et al. [17] reported a difference of 43% versus 29.5% uptake in groups invited by telephone calls and letters, respectively, in Luton. Uptake was even higher when face-to-face invitations were used (71.9%), which is akin to in-practice opportunistic invitations; a method that is relatively widespread, but not well measured or reported. However, an unpublished study in Bristol exploring the efficacy of a telephone outreach service to invite individuals from deprived areas observed lower overall uptake in general practices that used the telephone calls compared with non-telephone practices (24 vs. 36%) [18].

Despite some evidence that telephone invitations are associated with higher levels of uptake, they are rarely adopted by general practices. This might be a result of the perceived demands on practice staff time and the associated costs. Consequently, there has been work to design invitation letters to increase uptake at no extra cost. Sallis et al. [19] reported a modest, but significant effect when using a letter informed by behavioural insights compared with the previous national standard letter (33.5 vs. 29.3%, OR = 1.26, 95% CI 1.09–1.47). The improved letter was simpler and shorter (than the previous version), included the instruction to call to book an appointment (behavioural specificity), aimed to increase the personal salience (‘you are due to attend your NHS Health Check’) and had a tear off slip to record the date, time and location of the appointment to address the intention-behaviour gap. A similar study found the same improved letter, modified to include a deadline (i.e., ‘your Health Check is due in August’) also reported uptake that was 3 percentage points higher than the previous national template (18 vs. 21%) [20]. This improved invitation letter has since been adopted as the national template (and was used as the control invitation method in the present study).

We explored the use of tailored risk messages to improve postal invitations, making them personally tailored to patients’ level of CVD risk to increase their motivation to attend. Tailored risk messages have been shown to be more relevant to individuals, resulting in better processing, perception and understanding [21, 22]. A number of systematic reviews have looked into the effects of tailored information on behaviours in screening programmes. Personalised risk information has been found to increase realistic and more accurate perception of risk, improve knowledge, and increase uptake in comparison to general information [23, 24]. The way information about risk is presented can also impact on individual’s decision making [25]. Results suggested that the most effective presentation of risk incorporates relative risk information (rather than absolute risk) and using ‘loss framing’ (rather than ‘gain framing’). In a review of individualised risk communication by Edwards et al. [24], it was concluded that patients are able to make more informed decisions regarding screening tests when presented with risk information that is relevant to their own personal risk as opposed to general population risk.

We report findings from HEalth Check TRial (HECTR), a randomised controlled trial (RCT) to test whether invitation letters personalised to patient’s CVD risk could elicit higher uptake than the current national template letter, and also to compare with uptake in response to telephone invitations. A secondary aim was to use the relative costs of letter and telephone invitations to explore cost-benefit.

## Methods

### Aim

Our aim was to compare uptake of NHS Health Check in response to three different invitation methods: standard national letter, telephone invitation, and CVD risk-personalised letter.

### Study design and setting

A three-arm randomised controlled trial (RCT) was conducted across nine general practices in Stoke-on-Trent and Staffordshire, with individual patient randomisation. Ethical approval was received from the NHS Research Ethics Service Committee East of England – Cambridge (ref 15/EE/0340).

### Participants and recruitment

To be eligible, practices were required to: already be conducting NHS Health Checks; use the Egton Medical Information Systems (EMIS) practice software system (for compatibility with the trial processes); already use Health Check invitation methods that include letters and telephone calls (to minimise changes or additions to routine practice); and make sufficient Health Check invitations over a 12-month period to meet sample size requirements.

Practice recruitment involved several stages. First, all practices in Stoke-on-Trent and Staffordshire that used EMIS were emailed with information about the trial, requesting expressions of interest from Practice Managers. Expressions of interests were received from 45 practices (~ 35%). Second, all were followed up by telephone to discuss trial requirements, assess eligibility and confirm interest. Third, subsequent visits to meet Practice Managers were conducted in 15 practices considered willing and eligible to meet the trial requirements, with minimal changes or additions to current practice. Of these, the 10 most suitable were selected to participate (anticipated numbers of Health Check were too low in those excluded) and one was later excluded as they were unable to meet the trial requirements. Table 1 summarises characteristics of the nine practices included.

**Table 1:** Summary of practice-level information

	N	%
Practice		
Size		
Small (< 8000)	4	44.4
Large (≥8000)	5	55.6
Deprivation (where Q1 = most deprived)		
Q1	1	11.1
Q2	2	22.2
Q3	0	0.0
Q4	5	55.6
Q5	1	11.1
Letter invitations		
Practice staff responsible		
Data quality	3	33.3
Admin or PM	5	55.6
HCA/PN	1	11.1
External printing/postage		
Yes	4	44.4
No	5	55.6
Telephone invitations		
Practice staff making calls		
Amin	6	66.7
HCA/PN	2	22.2
Other	1	11.1
Calls outside usual work hours		
Yes	7	77.8
No	2	22.2
Health Checks		
Offered outside usual work hours		
Yes	5	55.6
No	4	44.4
Specific clinics		
Yes	3	33.3
No	6	66.7

*Q* Quintile, *PM* Practice Manager, *HCA* Health Care Assistant, *PN* Practice Nurse

Within each practice, patients were eligible if they met the national Health Check eligibility criteria [26] and were due to be invited during the 12-month trial.

### Procedures

To allow practices to follow trial procedures, EMIS provided practices with a detailed workflow/specification document. This provided instructions on searches, queries and read codes necessary to identify the eligible cohort as ‘trial participants’, for random allocation, and coding of various exclusions or end-points. EMIS also created a bespoke template to be used in conjunction with the specification document, aiming to standardise trial processes across practices.

Within practices, eligible patients were identified through EMIS searches and randomly allocated to the three trial arms: standard letter (SL), telephone (TP), risk-personalised letter (PL). In each arm, patients could be invited up to three times before being classified as a non-attender. They were also classified as a non-attender if they declined the invitation or responded, but failed to attend their Health Check appointment.

Patients were excluded from analysis if they attended a Health Check as the result of an opportunistic invitation, had invalid contact details to allow an invitation (telephone number in the TP arm; postal address in SL and PL arms), where there were known practice errors, or if the practice had not attempted to contact them.

The trial was planned to run in each practice for 12 months or until the eligible patient list for each arm/practice was exhausted. Due to practice-level delays in Health Check invitations, practices ran the trial for 12–15 months (between Dec-15 and Feb-17).

### Trial arms

#### Standard letter (SL, control group)

The current national template was used as the standard letter (or control). As detailed earlier this was developed using behavioural insights to improve the previous national template [19]. The current national NHS Health Check leaflet accompanied the letter. Up to three letters were sent to each patient before they were classified as a non-attender.

#### Telephone calls (TP)

Practice staff, predominantly reception/administrative staff or the Practice Nurse or Health Care Assistant, made telephone calls to invite patients (Table 1). To standardise the type of information relayed and provide prompts to likely questions, a guide / script was provided (Additional file 1). This was developed with input from practice staff. Specific training in how to conduct telephone invitations was not necessary as all participating general practices had experience of using telephone calls for NHS Health Check invitations (one of the eligibility criteria). However, practices were free to request further guidance regarding calls. Up to three attempts were made to invite patients, leaving messages where possible, and excluding patients if the telephone number was missing or incorrect.

#### Risk-personalised letters (PL)

The risk-personalised letters were developed to include messages appropriate for different levels of CVD risk based on patient’s % 10-year risk score (QRISK®2, [29]). Three letter templates were developed according to risk category: high ≥20%; medium 10–19.9%; low < 10%. The letter development phase involved several steps. First, working with the Public Heath England (PHE) Behavioural Insights Team, provisional templates for risk-personalised letters were developed from the national template. Second, these were shared with members of the HECTR steering group and subject experts for comment. Third, they were tested with the general public through a paper-based survey distributed at a number public settings (e.g., Libraries, Council offices) and using an online Qualtrics survey distributed to local workplaces and networks (*n* = 335). The survey estimated participant’s level of CVD risk based on existing chronic conditions or health issues (e.g., hypertension, high cholesterol, diabetes) and assigned the appropriate personalised letter. It then asked a series of questions about the letter (e.g., ease of understanding, emotional response) using a 7-point Likert scale and provided the opportunity for additional feedback. Survey data indicated that the risk-personalised letters were generally easy to understand (median of 6 = ‘very easy to understand’), did not make them feel excessively worried (median of 2 = ‘not really’ worried), nor panicky (median of 1 = ‘not at all’ panicky).

For patients allocated to the personalised letter arm, EMIS generated their % 10-year CVD risk score based on a range of indicators (e.g., age, gender, smoking status, systolic blood pressure, cholesterol). Where QRISK®2 score was missing, the system provided an estimated score based on the information present. For each variable used to generate the risk score including blood pressure and cholesterol, QRISK uses the most recent patient data available and, if there are missing or incomplete data, the score is estimated and the assumptions are shown in the system. Based on this score, EMIS allocated the appropriate letter (High, Medium, Low) to patients in the PL arm.

The current national NHS Health Check leaflet accompanied risk-personalised letters. Up to three letters were sent to each patient before they were classified as a non-attender.

### Measures

The primary outcome was attendance at an NHS Health Check (binary measure). Other patient-level data extracted from patient records included: age, gender, ethnicity, Lower Super Output Area of their home neighbourhood (to derive deprivation [27] and urban-rural classification [28]), % 10-year CVD risk (QRISK®2, [29]), and height and weight to determine Body Mass Index (BMI, kgm^− 2^). Practice-level information included: practice size (number of registered patients), staff responsible for Health Check invitations (letter and telephone calls), whether any telephone invitations were made outside of usual working hours, whether Health Check appointments were available outside usual working hours, and if Health Checks were organised as specific clinics or ad hoc.

To allow basic cost-benefit analysis, we collected data from each practice on the estimated resource required to administer the invitations by letter (cost of printing and postage) and telephone calls (estimated time per call and hourly salary of the staff making calls). Estimated costs of printing and postage per letter ranged from £0.54 to £3.40 (mean £2.39 ± £1.42). As some practices were considered to over-estimate these costs (e.g., £2per printed letter), we used costs reported by the four practices that used external companies for postage/printing, which appeared to be the most economical approach (mean £0.61 ± 0.07 per letter). For personalised letters, we deemed that the cost of practice staff time needed for the one-off process of adding the EMIS template to generate risk-personalised letters was negligible (approx. £20 one-off cost). The cost of a telephone invitation was estimated at £0.73 (mean hourly pay for relevant staff of £8.86/h * mean call duration of 5.1 min). We assumed an average of two invitations per patient to estimate the total cost per patient of £1.22 for letters and £1.46 for telephone invitations.

### Sample size

Local Health Check targets for 2014/2015 (Stoke-on-Trent) informed the original sampling, from which we estimated a mean practice target of 250 completed Health Checks over 12 months. Across 10 practices this equated to 2500 participants, or 833 per treatment arm. Using binary logistic regression with the proportion of the sample of 0.502 showing the target behaviour (50.2% attending health check, the local uptake rate at the time of designing the trial), a sample of 2500 would yield power of 0.8 with an odds ratio of 1.12 [30]. It is likely that effect size would need to be larger to achieve that level of power to take account of the relationships between the predictor variables and in particular how much of the variance in experimental condition was explained by the other predictor variables. At the design stage this was an unknown quantity. As an illustration, if 25% of variance in experimental condition could be accounted for by the other predictors (a large effect size), then, with the same sample size, the effect size would have to be an odds ratio of 1.14 for the analysis to have the same level of power. For a two-level logit analysis, assuming an intraclass correlation of 0.1 [31], the effect size would have to increase to an odds ratio of 1.92 to have the same level of power, with the same sample size [32]. We, therefore, took a pragmatic approach of running the trial for 12 months, whereby practices were asked to invite the entire eligible cohort for that year, which would need to be in excess of the target number of attended Health Checks. This approach aimed to minimise changes to practice, whilst allowing for the lower participant numbers in smaller practices and for exclusions.

### Statistical analysis

Characteristics of patients in each trial arm were compared using chi-squared tests to identify between-group differences. Logistic regression was used to identify the effects of invitation method on the likelihood of Health Check attendance (binary outcome: 0 = non-attender, 1 = attender). We first ran a single-level model and then explored practice effects on attendance using the likelihood ratio test statistic. As this confirmed practice effects (166.01; *p* < .001) we fitted a two-level logit model (level 1-individual, level 2-practice). Models were adjusted for a range of factors at the individual-level (age, gender, deprivation level, urban-rural residence, 10-year % CVD risk) and practice-level (practice size, telephone calls made outside usual working hours, offer of Health Check appointments outside of working hours). Ethnicity is reported descriptively, but was not included in regression analysis because: data were missing for 914 patients (19.8%); inconsistent coding across practices and small numbers of patients in non-White British ethnic categories necessitated a dichotomous variable; the dichotomised variable was not a predictor of uptake.

Cost-benefit was explored in two ways: relative cost per attended Heath Check was estimated using the basic percentage uptake figures (% uptake as a proportion * cost of invitation per patient); the additional cost per 1000 patients of using telephone invitations (£0.24*1000) or personalised letters (£0.00*1000) compared with standard letters, were considered in relation to the relative number of patients (per 1000) who would be expected to attend when invited by telephone invitations or personalised letters, compared with standard letters (marginal effect*1000).

## Results

### Sample characteristics

**Fig. 1:**
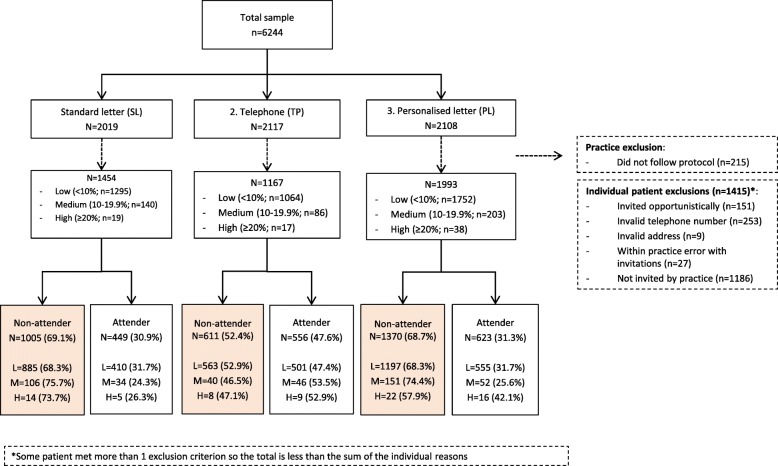
Trial flow diagram

The flow of patients through the trial and exclusions are summarised in Fig. 1. Patient characteristics overall and by trial arm are displayed in Table 2. In total, 6244 patients across 10 general practices were randomised to the three trial arms (SL *n* = 2019, TP *n* = 2117, PL *n* = 2108) of which 1630 were excluded: one practice was unable to invite sufficient patients to meet the trial demands (*n* = 215); 1186 were not invited by the remaining practices; 229 patients were excluded for other reasons (Fig. 1). This left a final sample of 4614 (SL *n* = 1454, TP *n* = 1167, PL *n* = 1993) across nine practices.

**Table 2:** Sample characteristics by trial arm

	Standard letter		Telephone		Personalised letter		Total		Difference	
	n	%	N	%	n	%	n	%	χ^2^	df
Total	1454		1167		1993		4614			
Age										
40–54	1108	76.2	896	76.8	1462	73.4	3466	75.1	16.00***	2
55–64	224	15.4	207	17.7	382	19.2	813	17.6		
65–74	122	8.4	64	5.5	149	7.5	335	7.3		
Missing	0		0		0		0			
Gender										
Male	713	49.0	534	45.8	948	47.6	2195	47.6	2.79	1
Female	741	51.0	633	54.2	1045	52.4	2419	52.4		
Missing	0		0		0		0			
Ethnicity										
White British	1100	93.9	915	95.1	1461	93.3	3476	93.9	3.49	1
Non-WBRI	72	6.1	47	4.9	105	6.7	224	6.1		
Missing	282		205		427		914			
BMI (kg/m^2^)										
Healthy range	480	33.0	405	34.7	653	32.8	1538	33.3	3.77	3
Overweight	458	31.5	375	32.1	660	33.1	1493	32.4		
Obese	312	21.5	240	20.6	380	19.1	932	20.2		
Morbidly obese	39	2.7	34	2.9	58	2.9	131	2.8		
Missing	165		113		242		520			
CVD risk										
Low	1295	89.1	1064	91.2	1752	88.0	4111	89.1	9.51	2
Moderate	140	9.6	86	7.4	203	10.2	429	9.3		
High	19	1.3	17	1.5	38	1.9	74	1.6		
Missing	0		0		0		0			
Deprivation (quintile)										
1 (most deprived)	226	15.5	180	15.4	379	19.0	785	17.0	39.72***	4
2	285	19.6	233	20.0	469	23.6	987	21.4		
3	322	22.1	233	20.0	406	20.4	961	20.8		
4	461	31.7	410	35.1	524	26.3	1395	30.2		
5	160	11.0	111	9.5	213	10.7	484	10.5		
Missing	0		0		2		2			
Area morphology										
Urban	1121	77.1	851	72.9	1571	78.9	3543	76.8	14.88***	1
Rural	333	22.9	316	27.1	420	21.1	1069	23.2		
Missing	0	0.00	0	0.00	2	0.10	2	0.04		

BMI, Body Mass Index; *** *p* < 0.01

Sample mean age was 50.2 ± 8.0 years and 75% were in the youngest age category (40–54 years). This was a reflection of the timing of the study (see Discussion). There were slightly more women than men. The majority were classified as having White British ethnic background and were urban dwellers. There was relatively good representation across the deprivation quintiles (Table 2). The majority of the sample (89%) were classified as ‘low CVD risk’ (< 10%).

Comparisons between trial arms highlighted statistically significant differences in terms of age, deprivation and urban-rural residence (Table 2). These were not a concern given the magnitude of differences and because these factors were included in adjusted regression models.

### Practice-level characteristics

Average practice size was 8671 ± 3158, with four practices classified as small (< 8000 patients) and five large (≥8000). There was some variation in Health Check processes among practices (Table 1). Administrative staff were most often responsible for sending/generating invitation letters, followed by data quality staff. Four practices used external providers for printing/posting (DocMail, imail). For telephone invitations, administrative staff were most often responsible for making calls, followed by those delivering the Health Check. Most, but not all practices, made some calls outside of usual working hours. In terms of the Health Check appointments, five out of nine practices offered them outside usual working hours and three ran dedicated Health Check clinics.

### Effect of invitation method on attendance at the NHS HC

In total, 1628 (35.3%) patients attended their Health Check (30.9% SL vs. 47.6% TP vs. 31.3% PL; Fig. 1). Unadjusted chi-squared analysis confirmed that, compared with those invited by the standard letter, uptake was significantly higher in patients invited by telephone (χ^2^ = 76.95, *p* < .001), but not by risk-personalised letter (χ^2^ = .056, *p* = .812).

From the single-level logit model, we found evidence that the likelihood of attending a Health Check was 16.2 percentage points higher for patients in the telephone arm compared with those in the standard letter arm, but with no significant effect for the personalised letter arm (Table 3).

**Table 3:** Marginal effects on likelihood of attending Health Check from single-level and multi-level logistic regression

Predictor variables	Marginal effects	
	Single-level	Multi-level
Invitation method *(ref. standard letter)*		
Telephone	.162***	.180***
	(.0190)	(.0374)
Personalised letter	.0118	.0400**
	(.0173)	(.0182)
Age (years)	.00660***	.00611***
	(.00144)	(.00151)
Gender	.0434***	.0443***
*(male = 0, female = 1)*	(.0157)	(.0164)
Deprivation quintile	.0441***	.0202**
*(1 = most to 5 = least deprived)*	(.00633)	(.00843)
Area morphology *(urban = 0, rural = 1)*	−.0149	−.0357*
	(.0184)	(.0195)
CVD risk (% 10-year score)	−.00854***	−.00738***
	(.00270)	(.00270)
Practice size	7.30e-06***	3.17e-06
	(2.69e-06)	(1.38e-05)
Health Checks outside working	−.0375**	−.0828
hours *(no = 0, yes = 1)*	(.0148)	(.0786)
Telephone invitations outside working	−.108***	−.0685
hours *(no = 0, yes = 1)*	(.0194)	(.0973)

Standard errors in parentheses

*** *p* < .01, ** *p* < .05, * *p* < .1

In the two-level logit model (accounting for practice effects), the likelihood of attendance was 18.0 percentage points higher in the telephone versus standard letter arm. Moreover, the likelihood of attendance was 4.0 percentage points higher in patients receiving the personalised letter, compared with the standard letter, independent of all confounders (Table 3).

Multi-level regression also confirmed some expected patterns, such as a significantly higher likelihood of attendance with increasing age, in women compared with men, and in residents of less deprived areas, but a reduced likelihood of attendance as CVD risk increased.

**Fig. 2:**
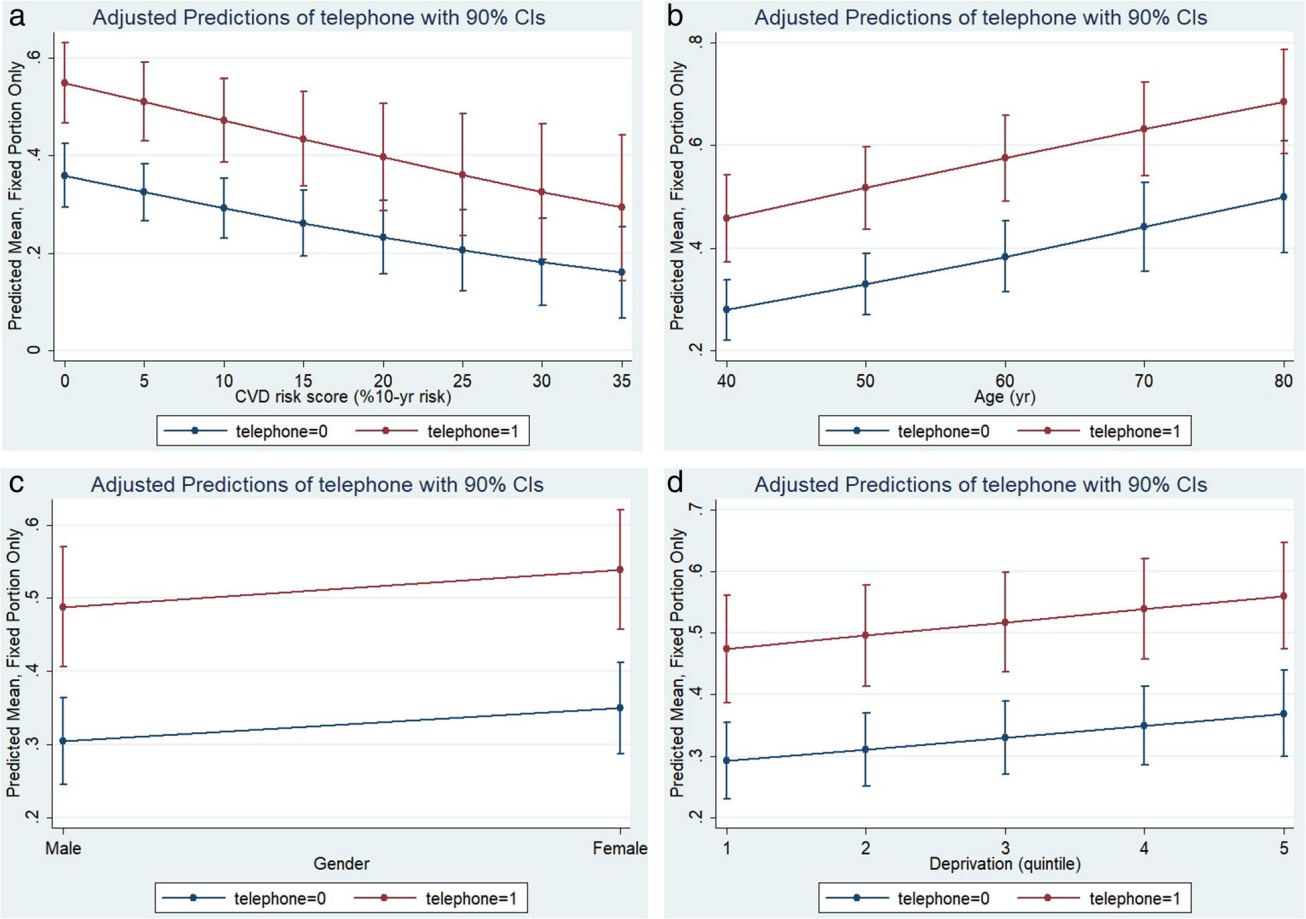
Adjusted predictions of uptake in telephone versus standard letter group by (**a**) CVD risk score, (**b**) age, (**c**) gender, (**d**) deprivation

Finally, by using the multi-level regression estimates, we derived predictions for the outcome variable of attendance across trial arms and patient characteristics. Telephone invitations were more effective than standard letters for patients with low levels of CVD risk and for those at the younger end of the age range. However, telephone invitations did have differential effectiveness compared with standard letters by gender or deprivation (Fig. 2a-d).

### Cost-benefit

Using the basic percentage uptake figures (Fig. 1), a cost of £1.22 per patient for letters and £1.46 for telephone calls equated to invitation costs per attended Health Check of £3.95 for standard letters, £3.90 for personalised letters and £3.07 for telephone invitations.

Using the marginal effects from the adjusted multi-level model for personalised letters (.400) and telephone invitations (0.180) (Table 3), we estimated the cost-benefit per 1000 patients invited relative to standard letters. For every 1000 patients invited using personalised letters (vs. with standard letters), an additional 40 Health Checks would be expected, at no extra cost. For every 1000 patients invited by telephone (vs. standard letters), an additional 180 attended Health Checks would be expected, costing an extra £240 (£0.24/patient).

## Discussion

### Main findings

The HECTR study was a three-arm RCT in 4614 patients across nine general practices in Stoke-on-Trent and Staffordshire to compare uptake of NHS Health Checks in response to different invitation methods. In adjusted multi-level regression, the likelihood of attendance was significantly higher when patients were invited by telephone, rather than by the current national health check invitation letter (18.0 percentage points). There was a smaller, but statistically significant effect for the risk-personalised letter, whereby attendance was 4 percentage points higher (compared with the standard letter). The relatively stronger effect of telephone invitations on uptake in those at with lower levels of CVD risk and at the younger end of the age range for Health Checks indicated that these characteristics could be used for targeting of telephone invitations to maximise effectiveness.

Cost data indicated that telephone invitations were more expensive than letters, but that this additional cost was modest (24p per patient) and justified given the expected 18 percentage point increase in attendance. To put this in context using local figures, for the 23,682 patients in Stoke-on-Trent and Staffordshire who were offered a NHS Health Check in 2017–2018 [33], telephone invitations could deliver 4376 additional completed Health Checks at an additional cost of £5835, whereas risk-personalised letters could result in 947 additional Health Checks at no extra cost.

### What is already known

Uptake of NHS Health Checks is below the required level and method of invitation has been identified as a strong predictor of uptake. Letters are the most commonly used, but apparently ineffective method of Health Check invitation [11, 17]. Qualitative data have highlighted a lack of impact of letters and perceived lack of personal relevance in people who do not respond to NHS Health Checks invitation letters [16]. Amendments to NHS Health Check letters using behavioural insights can elicit modest improvements (3–4 percentage points) in areas with relatively low baseline levels of uptake (18–31%) [19, 20]. Tailoring messages to individual’s level of risk can be beneficial for uptake in screening programmes [23, 24], but this had not been explored for NHS Health Check. The closest study (published after HECTR commenced) was a trial in which intervention participants were posted a Question-Behaviour-Effect (QBE) questionnaire focused on thoughts and feelings about attending an NHS Health Check before receiving the standard invitation letter [34]. The authors found no evidence of impact, but were limited by the response rate (23% returning the questionnaire).

Telephone invitations have been linked with higher levels of uptake, but are not widely used [17]. This could be a consequence of the perceived demands on staff time to make calls and, perhaps, because practices tend to be incentivised on the number of completed health checks, not percentage uptake [35]. Public Health England’s ‘*Top tips for increasing the uptake of NHS Health Checks*’ do not currently include telephone invitations as a strategy [36]. They include text message reminders and targeted telephone outreach (in which part of the Health Check is completed over the phone), but the latter is described as labour and cost intensive.

### What this study adds

Our data provide evidence that telephone invitations should be used as a way to improve uptake of NHS Health Check without incurring much additional cost and could be targeted based on age and estimated CVD risk. Cardiovascular disease risk-tailored letters could offer further modest improvements on the current behavioural insights-informed national template [19]. Given the substantial deficit between current and required levels of uptake nationally (~ 50 versus 75%), the salient finding from HECTR is that telephone calls are considerably more effective and justify the additional cost. It is possible that our cost-benefit of telephone versus letter invitations over-estimated the additional cost of telephone calls as: we used the lowest practice estimates of postal invitation costs and did not include the staff time to receive calls from patients responding to invitation letter to book their Health Check after receiving a letter (a process that would typically happen as part of the telephone invitation call).

On the basis of HECTR, we recommend that telephone invitations are advocated as an efficient strategy to improve NHS Health Check uptake, particularly in younger and lower CVD risk individuals. We recommend that others explore the relative cost of postal versus telephone invitations to confirm those estimated by HECTR practices. Should this confirm our findings, work might be required to challenge primary care staff perceptions around the cost-benefit to practices of NHS Health Check telephone invitations and to incentivise general practices to improve uptake.

### Limitations

A number of limitations are recognised. First, for reasons beyond our control, the trial commenced at a time when many Stoke-on-Trent and Staffordshire general practices were reaching the end of the five-year NHS Health Check cycle (during which all eligible patients should be invited). Patients yet to attend a Health Check are more likely to belong to the ‘harder to reach’ groups who are less likely to engage in primary prevention programmes. For NHS Health Check, this includes people at the younger end of the age range. This was confirmed as 75% of our sample were aged 40–54 years. As QRISK®2 is heavily influenced by age, 89% were classified as low risk. Consequently, relatively small numbers in the personalised letter arm received the medium and high risk-tailored letters. Second, most participating practices were slow to complete up to three invitations for their eligible cohort. Many did not invite all patients who were randomised, which reduced the overall sample size (*n* = 1186 not invited and one practice with 215 eligible patients was excluded; Fig. 1). Third, the EMIS template (designed for this study) coded telephone calls as ‘failed to respond’ rather than recording each call made. This reduced the reliability of data regarding the number of telephone calls made per patient and prevented meaningful analysis of non-responders (i.e., those who did not respond to the call). Analysis therefore, focused only on the primary outcome of attendance versus non-attendance, and did not allow exploration of response versus non-response to invitations. Fourth, our nine general practices did not provide the number of clusters preferable for multi-level analysis (≥20). However, significant inter-practice variation reported in other studies of NHS Health Check uptake [12, 37] was confirmed by the likelihood ratio test. Fifth, as fidelity of the telephone invitations was not measured, variation in approach between staff and practices is likely. Finally, HECTR was limited to one county so generalisability to all areas of the country cannot be assumed.

## Conclusions

Our findings support the use of telephone invitations to improve uptake in NHS Health Check, especially in those at the younger end of the target age range and those with lower CVD risk. Uptake achieved through generic letters alone is not sufficient. Risk-tailoring appears to offer a modest improvement on the current national template letter, but this should be explored further in a larger sample of high risk individuals and using other metrics, such as heart age, that are less age-dependent. Further work should be undertaken to confirm the considerable benefit and relatively small additional cost of telephone invitations.

## Additional file

Additional file 1: Telephone invitation guide. Guide / script provided to general practices to use when making telephone invitations. (DOCX 28 kb)

## Abbreviations

BMI: Body Mass Index
CVD: Cardiovascular disease
EMIS: Egton Medical Information Systems
HECTR: HEalth Check TRial
PHE: Public Heath England
PL: Risk-personalised letter
QBE: Question-Behaviour-Effect
RCT: Randomised controlled trial
SL: Standard letter
TP: Telephone
