# Supplementary material for: Randomised controlled trial comparing uptake of NHS Health Check in response to standard letters, risk-personalised letters and telephone invitations
Source: BMC Public Health. 2019 Feb 21;19:224. doi: 10.1186/s12889-019-6540-8 (PMC6385450; doi:10.1186/s12889-019-6540-8)
Supplement: Supplementary file 1 — Telephone invitation guide. Guide / script provided to general practices to use when making telephone invitations. (DOCX 28 kb) [file 12889_2019_6540_MOESM1_ESM.docx]

Additional file 1: Telephone invitation guide

Hello Mr/Mr/Mrs [XXXXX], this is [XXXX] from [XXXXX] doctor’s surgery.

I am calling to invite you for your free NHS Health Check. Patients aged between 40 and 74 are entitled to a free NHS Health Check to assess their risk of developing heart disease, stroke, kidney disease or diabetes. If there are any warning signs, then together we can do something about it. Would you be interested in a free NHS Health Check?

I am calling to invite you for your free NHS Health Check.

Have you heard of NHS Health Checks?

If **yes or no**: These checks are being offered to people aged between 40 and 74 once every five years. The aim is to assess your risk of developing heart disease, stroke, kidney disease or diabetes. If there are any warning signs, then together we can do something about it.

The Benefit of a Health Check is that if any risk is identified, we can take early action, improve your health and prevent the onset of these conditions. There is good evidence for this.

What is involved: The check should take about 20–30 minutes and is based on straightforward questions and measurements such as age, sex, family history, height, weight and blood pressure. You will also need a simple blood test to measure your cholesterol level.

Following the check, you will receive free personalised advice about what you can do to stay healthy.

Can I book you in for an appointment?
